# Cl-Assisted Large Scale Synthesis of Cm-Scale Buckypapers of Fe_3_C-Filled Carbon Nanotubes with Pseudo-Capacitor Properties: The Key Role of SBA-16 Catalyst Support as Synthesis Promoter

**DOI:** 10.3390/ma10101216

**Published:** 2017-10-23

**Authors:** Filippo S. Boi, Yi He, Jiqiu Wen, Shanling Wang, Kai Yan, Jingdong Zhang, Daniel Medranda, Joanna Borowiec, Anna Corrias

**Affiliations:** 1 College of Physical Science and Technology, Sichuan University, Chengdu 610064, China; 2015521221002@stu.scu.edu.cn; 2 Analytical & Testing Center, Sichuan University, Chengdu 610064, China; scu_heyi@126.com (Y.H.); wenjiqiu@scu.edu.cn (J.W.); wangshanling@scu.edu.cn (S.W.); 3 School of Chemistry and Chemical Engineering, Huazhong University of Science and Technology, Wuhan 430072, China; yank@hust.edu.cn (K.Y.); zhangjd@mail.hust.edu.cn (J.Z.); 4 School of Physical Sciences, University of Kent, Canterbury CT2 7NZ, UK

**Keywords:** carbon nanotubes, ferromagnetic Fe_3_C, SBA-16, buckypaper, capacitor

## Abstract

We show a novel chemical vapour deposition (CVD) approach, in which the large-scale fabrication of ferromagnetically-filled cm-scale buckypapers is achieved through the deposition of a mesoporous supported catalyst (SBA-16) on a silicon substrate. We demonstrate that SBA-16 has the crucial role of promoting the growth of carbon nanotubes (CNTs) on a horizontal plane with random orientation rather than in a vertical direction, therefore allowing a facile fabrication of cm-scale CNTs buckypapers free from the onion-crust by-product observed on the buckypaper-surface in previous reports. The morphology and composition of the obtained CNTs-buckypapers are analyzed in detail by scanning electron microscopy (SEM), Energy Dispersive X-ray (EDX), transmission electron microscopy (TEM), high resolution TEM (HRTEM), and thermogravimetric analysis (TGA), while structural analysis is performed by Rietveld Refinement of XRD data. The room temperature magnetic properties of the produced buckypapers are also investigated and reveal the presence of a high coercivity of 650 Oe. Additionally, the electrochemical performances of these buckypapers are demonstrated and reveal a behavior that is compatible with that of a pseudo-capacitor (resistive-capacitor) with better performances than those presented in other previously studied layered-buckypapers of Fe-filled CNTs, obtained by pyrolysis of dichlorobenzene-ferrocene mixtures. These measurements indicate that these materials show promise for applications in energy storage systems as flexible electrodes.

## 1. Introduction

For more than a decade carbon nanotubes (CNTs) have been considered as a fundamental focus of nanotechnology, thanks to their outstanding physical and electrical properties, which are strongly dependent on their chirality and curvature [1,2,3]. These nanostructures have attracted a great attention also for their exceptional chemical stability. The latter has been widely investigated not only for biomedical purposes [4], where CNTs have been considered ideal nanocapsules for the encapsulation of specific nanomedicine contrast agents [4], but also for numerous magnetic applications involving the encapsulation of a ferromagnetic material inside the carbon nanotube core [5,6,7,8,9,10,11,12]. The high chemical stability of CNTs ensures a complete protection of the encapsulated ferromagnet from the external environment and allows retaining the magnetic properties for long timescales. In this context, α-Fe and Fe_3_C have been widely studied owing to their promising magnetic properties, with high and tuneable saturation magnetizations and large coercivities [5,6,7,8,9,10,11,12]. These nanostructures are generally grown by chemical vapour deposition (CVD) of a metallocene (ferrocene) at high temperatures (approximately 1000 °C) in the form of vertically oriented films [5,6,7,8,9]. Despite the numerous reports on the control of the ferromagnetic filling rates of these vertically oriented nanostructures, one of the major problems that limit their translation into magnetic devices is the high brittleness. Indeed, CNTs-films produced using only ferrocene are generally very fragile, due to the low number of Van der Waals interactions and other imperfections in the vertical alignment. Recent works have shown that the stability of these structures can be improved through the addition of Cl-containing hydrocarbons, which allow the synthesis of CNTs-films with a higher number of Van der Waals interactions and less imperfections in the vertical alignment [13].

Despite the vast amount of work, the production of these nanostructures in large scale remains still challenging and strongly depends on the used substrate, on the evaporation temperatures of the precursors and on the chosen surface of growth [13]. Interestingly, recent reports have shown that another type of Fe-filled CNTs film, known as buckypaper, can comprise Fe-filled CNTs in the form of randomly entangled or horizontally aligned structures [14,15,16,17,18,19,20,21]. This type of film is generally synthesized directly in situ by CVD of ferrocene/dichlorobenzene or trichlorobenzene mixtures and can exhibit excellent magnetic properties and high elasticity [14,15,16,17,18,19,20,21]. Recent reports have shown that the morphology of these structures strongly depend on the used vapour flow rates, as well as on the chosen dichlorobenzene or trichlorobenzene concentration [14,15,16,17,18,19,20,21]. The excellent magnetic properties of these structures show a great promise for applications in electromagnetic devices, as well as in microwave absorption and aerospace technology [14].

However, some major challenges based on the control of purity and reaction efficiency remain. Indeed it has been shown that buckypapers grown in presence of Cl radicals at low evaporation temperatures generally present an unusual surface crust comprising empty and filled carbon nano-onions (CNOs) [13,14]. The presence of such an unusual crust therefore requires additional purification treatments that limit a direct use of this approach for industrial buckypaper production. In addition, the necessity of accurately control the concentration of dichlorobenzene in order to avoid the formation of metal chlorides represents a challenge limiting the growth of these nanostructures to only low precursor-evaporation temperatures and low Cl-concentrations [13,14,21]. Minimizing the quantities of dichlorobenzene could be considered a necessary step toward the large scale production of these types of carbon nanotubes films. New solutions are necessary for higher precursor-evaporation temperatures that are required for the future encapsulation of specific hard magnet alloys such as FePt, CoPt, Fe_5_Sm, and Co_5_Sm in buckypaper structures, given the high evaporation temperatures of the metal-containing metallocene-like precursors [22]. Up to now, high evaporation temperatures in the order of 200 °C can be used only for the production of vertically aligned CNTs structures, but not for the production of buckypapers [13].

In this context, the possible use of alternative supported-catalysts could be useful for promoting the growth of these nanostructured-films in a large scale. It has been shown that highly porous and high surface area matrices such as mesoporous ordered silicas can be considered as ideal candidates as support for nanoparticles to be used as catalysts for CNTs production [23,24,25,26,27]. These types of supported catalysts can allow for the synthesis of CNTs with a uniform diameter distribution, since the dimensions of the catalyst-nanoparticles are comparable to those of the mesoporous-silica pores. In particular, the so called SBA-16, which has a cubic arrangement of pores characterized by a body centered cubic symmetry (space group *Im*-3*m*) has attracted much attention [23,24,25,26,27], which stems from the spherical empty cages, with each cage connected to eight neighbouring one by narrow openings forming a 3D network of mesopores. Despite these promising characteristics, this type of supported catalysts has not yet been considered for promoting the growth of ferromagnetically filled CNTs buckypapers. 

In this work, we demonstrate that SBA-16 can have a crucial role in the fabrication of large-scale ferromagnetically filled buckypapers by favoring the growth of CNTs on a horizontal plane with random orientation rather than in a vertical direction. SBA-16 can therefore be considered not only as secondary catalyst source since it acts as catalyst support on the substrate surface but also as growth-inhibitor, since it can prevent the growth of CNTs in the form of vertically oriented films. The morphology and composition of the obtained CNTs-buckypapers was analyzed in detail by scanning electron microscopy (SEM), Energy Dispersive X-ray (EDX), transmission electron microscopy (TEM), high resolution TEM (HRTEM), and thermogravimetric analysis (TGA). Structural investigation of the buckypapers was performed by Rietveld Refinement methods of X-ray diffraction (XRD) data. The magnetic properties of the produced buckypapers were also analysed by vibrating sample magnetometry (VSM) at room temperature. Additionally, the electrochemical performances of these buckypapers are demonstrated and reveal a behavior that is compatible with that of a pseudo-capacitor with better performances than those presented in other previously studied layered-buckypapers of Fe-filled CNTs.

## 2. Materials and Methods

### 2.1. SBA-16 Synthesis

The synthesis of the SBA-16 support was performed according to reference [28]. Pluronic F127 (2.5 g) was dissolved in distilled water (120 g) and concentrated chloric acid (HCl, 5.25 g). After complete dissolution, n-butanol (C_4_H_10_O, 7.5 g) was added to this mixture at 45 °C. After one hour under stirring, tetraethyl orthosilicate (Si(OC_2_H_5_)_4_, 12.0 g) was added. The mixture was further stirred vigorously for 24 h and heated at 80 °C. The solid product was filtered, washed, and then calcined at 500 °C for 6 h with a heating step of 1 °C/min.

### 2.2. Supported Catalysts Synthesis

The SBA-16 supported catalyst was prepared by dissolving Sm(NO_3_)_3_ (0.6110 g, 1.375 mmol) and Fe(NO_3_)_3_ (2.774 g, 6.872 mmol) salts in 10 mL of deionized water. After complete salt dissolution, the SBA-16 (0.3 g) was added to the prepared mixture, followed by sonication (5 min), and subsequent stirring for 24 h at ambient temperature (29 °C). Finally, the product was collected by centrifugation (6 min, 8000 rpm) and redispersed in 8 mL of water.

### 2.3. Silicon Cleaning

The silicon wafers were cleaned with detergent, rinsed with deionized water, followed by sonication in water (5 min), acetone (2 times, 5 min), ethanol (2 times, 5 min), and isopropyl alcohol (2 times, 5 min). Finally, the wafers were dried in a flow of pure N_2_ gas (99.999%).

### 2.4. Supported Catalysts Spin Coating

3 mL of the suspension of the SBA-16 supported catalyst was spin coated on the pre-cleaned silicon wafers for 30 s at 1500 rpm, and the samples were then left to dry at ambient temperature (29 °C) for few hours.

### 2.5. Chemical Vapour Deposition Experiments

Mixtures of dichlorobenzene (0.15 mL) and ferrocene (1.5 g) were evaporated and pyrolyzed in a CVD system comprising a quartz-tube reactor length of 1.5 m (inner diameter of 44 mm, wall thickness of 3 mm), smooth Si/SiO_2_ substrates prepared according with the method described above (111 preferential crystal orientation, SiO_2_ thickness in the order of 90 nm and substrate dimensions of 6 cm length, 2.5 cm width and 0.525 mm thickness), and an electrical furnace set at the temperature of 990 °C. Note that the substrates were positioned in the region of the reactor where a temperature of approximately 900 °C is reached at the reaction stage. A pre-heater evaporation temperature of approximately 220 °C was used in these reactions, which is comparable with respect to that used in previous works on free-standing vertically aligned Fe_3_C-filled CNTs films grown in the presence of similar dichlorobenzene quantities [13]. An Ar flow rate of 11 mL/min was used in order to deliver the ferrocene and dichlorobenzene species in the reaction zone. The duration of the reaction was 1 h. The samples were cooled down using a quenching method by removing the furnace along a rail system.

### 2.6. Buckypaper Characterization

SEM images using backscattered electrons (B.E.) and EDX spectra were acquired using a JSM-7500F at 5–20 kV (JEOL, Tokyo, Japan). XRD patterns were acquired with an Empyrean Panalytical diffractometer (Cu K-α with λ = 0.154 nm) (PANalytical, Almelo, The Netherlands). Rietveld Refinement of the XRD pattern was done by using the software GSAS [29]. Cross-sectional TEM, HRTEM, EDX, and STEM analysis were performed with a 200 kV American FEI Tecnai G2F20 (FEI/ThermoFisher subsidiary, Hillsboro, OR, USA). The samples were prepared by dispersing the as grown buckypaper in ethanol. A copper grid with holey carbon film was then immersed in the ethanol-suspension with the use of magnetized twizers. The use of magnetized twizers is helpful for a fast deposition of the CNTs in the TEM grid. Thermal analyses were performed with a Mettler Toledo TGA/DSC 2/1600 Thermastar (Mettler Toledo, Zurich, Switzerland) under N_2_. The magnetic properties were investigated through a Quantum Design VSM at room temperature (300 K) (Quantum Design, San Diego, CA, USA).

### 2.7. Electrochemical Analyses

Potassium hexacyanoferrate (II) trihydrate (K_4_[Fe(CN)_6_]·3H_2_O), potassium hexacyanoferrate (III) (K_3_[Fe(CN)_6_]), and potassium chloride (KCl) were provided by Sinopharm Chemical Reagent Co., Ltd. (Shanghai, China). 

Electrochemical impedance spectroscopy (EIS) was performed on a CHI660A electrochemical workstation (Chenhua Instrument Co. Ltd., Shanghai, China) in a conventional three-electrode system. A modified electrode, a saturated calomel electrode (SCE) and platinum wire were employed as the working, reference, and counter electrode, respectively. EIS measurements were performed in 5 mmol L^−1^ K_3_[Fe(CN)_6_]/K_4_[Fe(CN)_6_] aqueous solution with 0.1 mol L^−1^ KCl as the supporting electrolyte, within the frequency range from 100 kHz to 0.1 Hz, and a bias potential of 0.2 V. The geometric surface area of glassy carbon electrode (GCE) was estimated to be 7 mm^2^, and that of CNTs/CNOs buckypapers and Fe_3_C/CNTs buckypapers electrodes to be 8 mm^2^.

## 3. Results and Discussion

**Figure 1:**
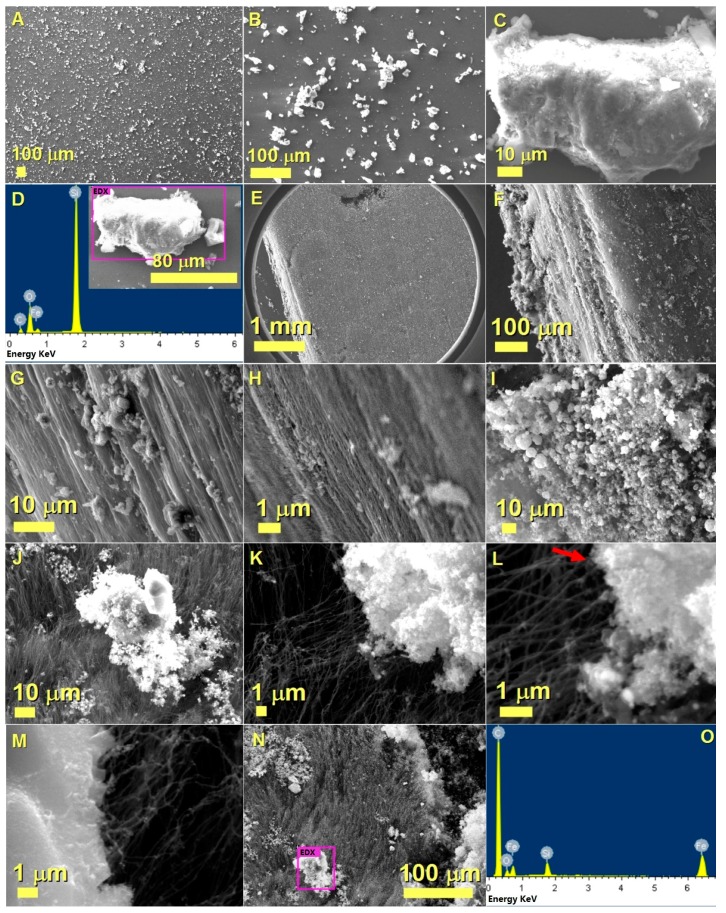
Scanning electron microscopy (SEM) images showing the Si/SiO_2_ substrate after deposition of the SBA-16 supported catalyst species from a top view in (**A**,**B**) and with a higher magnification in (**C**). In (**D**) the Energy Dispersive X-ray (EDX) spectrum obtained from the area shown in the inset is shown. The as grown buckypaper morphology and cross-section after chemical vapour deposition (CVD) are shown with an increasing level of detail from (**E**–**H**). In (**I**–**J**) the presence of the SBA-16 supported catalyst species is shown. In (**K**–**M**), SEM micrographs showing the presence of a direct connection between the grown carbon nanotubes (CNTs) and the SBA-16 supported catalyst are shown. In (**N**–**O**) a typical EDX analysis of the SBA-16 supported catalyst is shown.

The morphology of the as prepared substrate after spin-coating of FeSm/SBA-16 has been investigated by SEM, and the results are shown in Figure 1. As shown in Figure 1A,B, the catalyst supported on mesoporous silica appears to be uniformly distributed on the top of the Si substrate surface in the form of flakes. A higher magnification detail of a micrometre-size flake is shown in the image of Figure 1C. Typical EDX spectrum of the area displayed in Figure 1C is shown in Figure 1D (see also inset), which provided the following amounts of the elements present: 17.43 wt % of C, 31.85 wt % of O, 32.94 wt % of Si, and 17.78 wt % of Fe, corresponding to 29.42 at % of C, 40.36 at % of O, 23.77 at % of Si, and 6.45 at % of Fe. These results indicate that Fe-containing species are present within the SBA-16 flakes, while no Sm could be detected due to the very low quantities used in the synthesis-method described above.

The SEM morphological analysis performed on the as grown buckypaper after CVD are shown in Figure 1E–H. The cm-scale dimensions of the buckypaper were confirmed by the SEM image at low magnification (see Figure 1E). Further images of the buckypaper-cross-section obtained with an increasing magnification in Figure 1F–H revealed the thickness of the buckypaper in the order of approximately 290 μm. The buckypaper appears to be arranged in a layered like morphology, with each CNT-layer characterized by a random CNTs orientation in the horizontal plane. Note that as shown in Figure 1I–J, the catalyst particles together with the SBA-16 support were found on the top-surface of the analysed buckypaper. Analysis of this area at higher magnification revealed the presence a direct connection between the growing CNTs and the SBA-16 catalyst support. Such direct connection can be clearly observed in different regions of the analysed SBA-16 flake, as indicated in Figure 1K–M and in Figure 1L by the red arrow. 

Such direct connection between the grown CNTs and the supported catalyst suggests that SBA-16 has crucial role in driving the buckypaper growth by inhibiting the formation of vertically aligned CNTs, and instead favouring the formation of CNTs in the horizontal plane with random x-y directions in a spider-web like arrangement. In addition, the presence of SBA-16 on the top-region of the buckypaper suggests that after the first layer-growth is initiated, the other layers may grow one by one underneath the first one in a lift-up mechanism [21]. Note also that no onion-crust if found in the buckypaper-surface shown in Figure 1E,F. This suggests that the buckypapers produced in this work have a higher purity with respect to those reported in previous reports [13,14,21].

**Figure 2:**
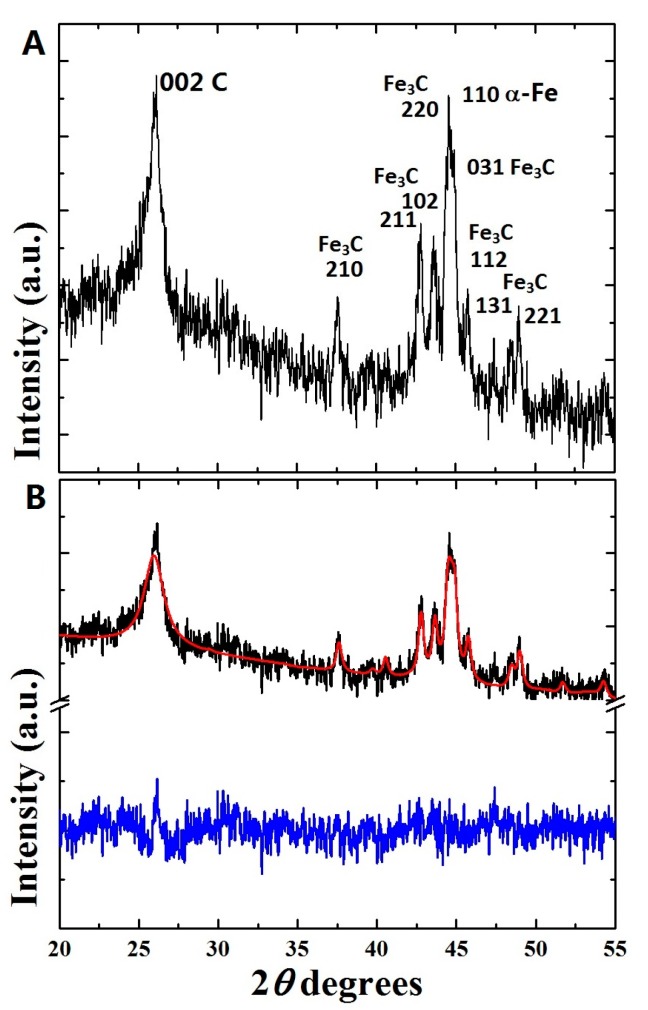
In (**A**) a typical X-ray diffraction (XRD) pattern of the as grown ferromagnetically filled CNTs buckypaper is shown. In (**B**) the XRD-pattern (black line) and Rietveld refinement (red line) of the as grown buckypaper is illustrated. The difference between the theoretical fit and the experimental data is represented by the blue line (**B**).

The presence of Si and O, together with Fe was confirmed by the EDX spectrum, shown in Figure 1O, derived from the region of the SBA-16 flake (Figure 1N). Instead, Sm could be detected only in separated regions in the form of micrometre sized particles, as highlighted in the following. Further characterization of the produced buckypaper was obtained using X-ray diffraction. In particular, the XRD pattern obtained from the analysed buckypaper is shown in Figure 2.

Interestingly, together with the 002 reflection arising from the CNTs-walls contribution, the 210, 211, 102, 220, 031, 112, 131, and 221 reflections due to the presence of Fe_3_C and an intense 110 reflection due to the presence of α-Fe were also detected. Instead, no clear reflection due to the γ-Fe phase was observed. Moreover, no peak is found due to Sm, which might be present in an amorphous phase. An amorphous halo detectable in the region of 25 degrees 2*θ* can be attributed to the presence of SBA-16 in the buckypaper.

The Rietveld method, which uses the least-squares approach to match a theoretical line profile to the diffraction pattern, was used to gather further information, such as identify and estimate the relative abundances of the phases present in the ferromagnetically filled CNTs buckypaper. As shown in Figure 2B, the Rietveld refinement confirmed the interpretation above with the following relative abundances of carbon (78.1%), α-Fe (2.5%), and Fe_3_C (19.3%). Furthermore, the following unit cell parameters were derived as follows: for Fe_3_C with space group Pnma a: 0.511 nm, b: 0.676 nm, c: 0.454 nm; for α-Fe with space group *Im*-3*m* a = b = c: 0.287 nm; for the carbon CNTs-walls graphitic contribution: a = b: 0.246 nm and c: 0.687 nm.

**Figure 3:**
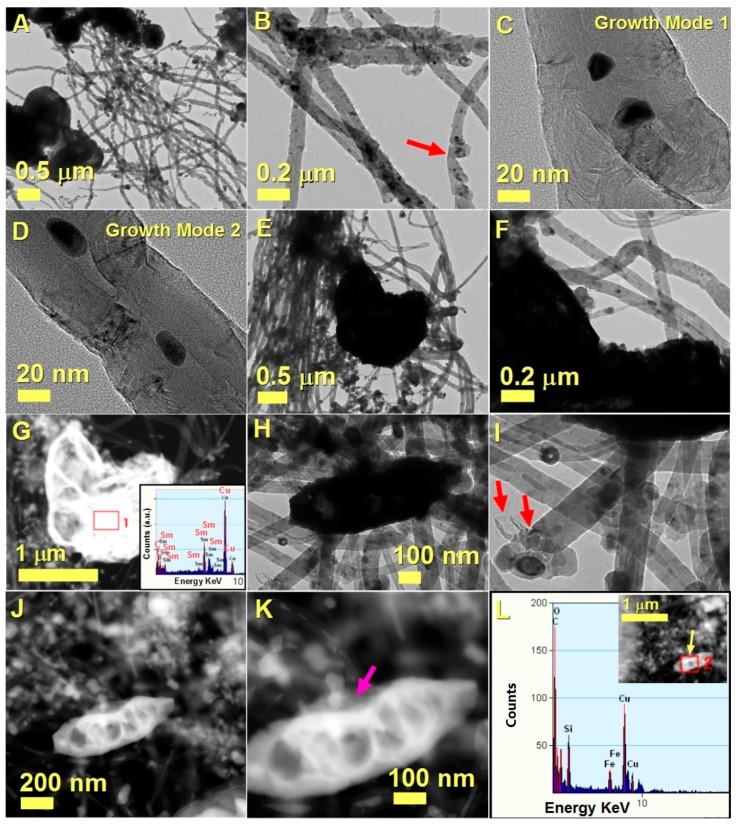
Transmission electron microscopy (TEM) (**A**–**F**) and high resolution TEM (HRTEM) (**C**,**D**) micrographs showing the cross sectional morphology of the CNTs comprised in the buckypaper. In (**G**), the STEM/EDX analysis of a micrometre-size catalyst particle showing the presence of both Sm and O (see inset) is shown. In (**H**–**K**) TEM (**H**,**I**) and STEM (**J**,**K**) micrographs showing the growth mechanism of a filled CNT departing from a SBA-16 flake are shown (see also magenta arrow in (**K**)). The red arrows in I show also the nucleation mechanism of another CNT detached from the SBA-16 support. The EDX analysis of the area in (**K**) is shown in (**L**) (see also yellow arrow in inset). See Supplementary Materials Figure S1 for statistical investigation of the CNTs diameter and for more information on the CNTs length.

The characterization of the CNTs present within the buckypaper was carried out by TEM, HRTEM and STEM/EDX, as shown in Figure 3. As shown in Figure 3A the analysed CNTs were found to be connected with micrometre-scale agglomerations of spherically encapsulated Fe-based catalyst particles. 

The presence of these particles can be considered a consequence of the in situ reduction of SBA-16 supported catalyst induced by the presence of high concentrations of hydrogen coming from the pyrolysed metallocene/dichlorobenzene vapours. Further analysis of the cross-sectional CNTs morphology also revealed the presence of two main growth mechanism (growth modes). A growth mode 1, in which the CNTs appear to grow in bamboo-like arrangement [30,31,32] (see red arrow in Figure 3B and HRTEM image in Figure 3C) and a growth mode 2 (see HRTEM image in Figure 3D), in which the CNTs appear to grow in a catalyst-pool like mechanism [13,14,32]. Note that in bamboo-like growth mechanisms, CNTs are generally characterized by the periodical repetition of closed compartments. Instead in the catalyst pool mechanism the presence of graphene caps is generally a consequence of the melted status of the catalyst particle during the CNTs growth and is indicative of the growth direction (the graphene cap is generally oriented toward the growth direction of the CNT) [13]. In addition, cross sectional EDX investigation in STEM mode allowed us to detect the presence of Sm in unusual thick micrometre size particles, as highlighted in the example shown in Figure 3G and in the inset reporting the EDX spectrum. Due to the high thickness, the information could not be obtained from the TEM and HRTEM images (Figure 3E,F). Instead, the EDX analysis in STEM mode revealed the compositional characteristics. Note that the high brightness is associated to the high Sm content within the particle. Curiously, the EDX spectrum did not evidence the presence of Fe within the particle, indicating that Fe and Sm did not alloy during the in situ reduction in the CVD process. In addition, the presence of oxygen suggests that an amorphous Sm_2_O_3_ phase is probably formed by exposure of the catalyst to air after the CVD experiment.

Further TEM images shown in Figure 3H,I also allowed for the direct observation of the cross-sectional morphology of the post-CVD SBA-16 matrix. A flake of SBA-16 with a platelet-like arrangement was found to be connected with numerous CNTs. An example of CNT detached from the flake is shown in Figure 3H,I. Note that many onion-like particles are connected to the CNT-base, however the nucleation of the CNTs appears to start from a not-encapsulated particle, as indicated by the red arrows. Further analysis of this area of the sample in STEM mode (see Figure 3J,K) also allowed for the observation of a CNT directly connected to the silica-based SBA-16 support. Note that, as shown in Figure 3K by the magenta arrow, the nucleation of the CNT appears to initiate directly from the SBA-16, which, as expected, is found to contain both silicon and oxygen (see compositional analyses in Figure 3L and area of analysis in Figure 3L-inset). These observations confirm the key role that SBA-16 plays in controlling the nucleation dynamics of the filled-CNTs in the buckypaper. 

**Figure 4:**
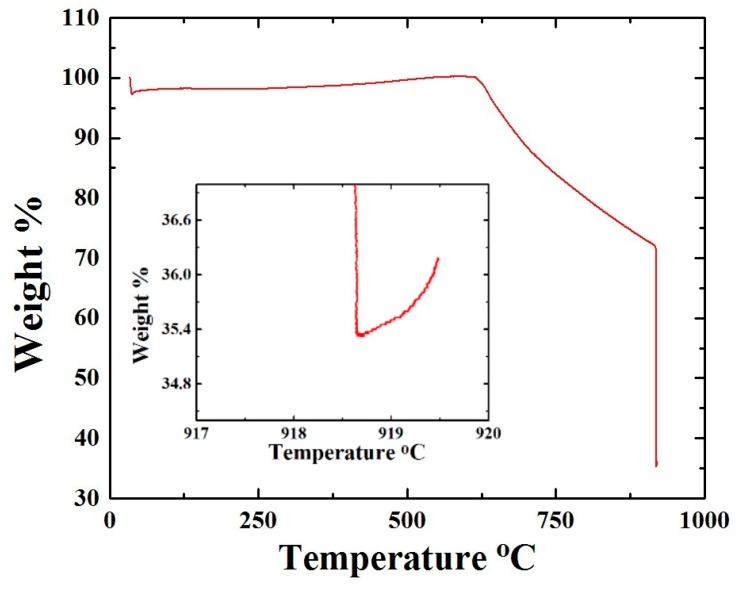
TGA analysis of the as grown buckypaper.

Further information on the composition of the grown buckypaper was obtained by thermogravimetric analysis (TGA) under N_2_, which was done in two steps: in step 1, temperature was increased up to approximately 919 °C, and, in step 2, the temperature was kept constant until complete decomposition. As shown in Figure 4, the results revealed the presence of approximately 64.6% of carbon, which could be attributed to the graphitic structure of the CNTs and 36.4% of metal, which can be attributed to both the Sm-based and Fe-based species within the buckypaper. 

**Figure 5:**
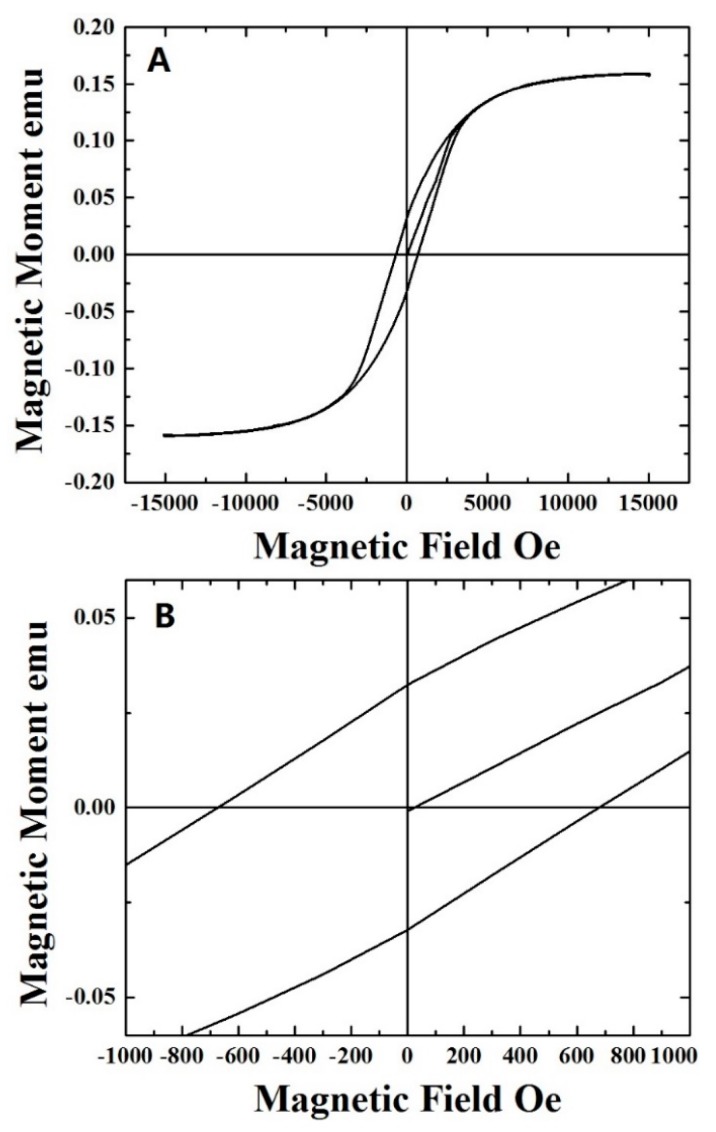
Room temperature magnetization versus field curves of the as grown CNTs buckypaper. (**A**) shows the typical ferromagnetic hysteresis loop of the sample; **(B**) shows the measured coercivity.

In order to measure the coercivity associated with the buckypaper the magnetic properties were measured at 300 K. As shown in Figure 5, a typical ferromatignetic-like behavior was found. A high coercivity, in the order of 650 Oe, was determined which is higher with respect to the values given in previous reports on cm-scale buckypapers filled with Fe_3_C [14,21]. Also, the observed coercivity is higher with respect to that of approximately 200 Oe reported by Rossella et al. [33] in the case of template grown cobalt cluster-filled CNTs arrays. The observed coercivity is however lower with respect to the values recently found in the case of other type of buckypaper morphologies filled with Fe crystals, due to the absence of geometry-induced exchange coupled interactions presented in that work [21]. Note also that the absence of γ-Fe in our sample also implies the absence of low-temperature antiferromagnetic phenomena. The magnetic properties are therefore much different with respect to those reported by Sahoo et al. in the case of antiferromagnetic Co_3_O_4_ nanoparticles [34]. 

**Figure 6:**
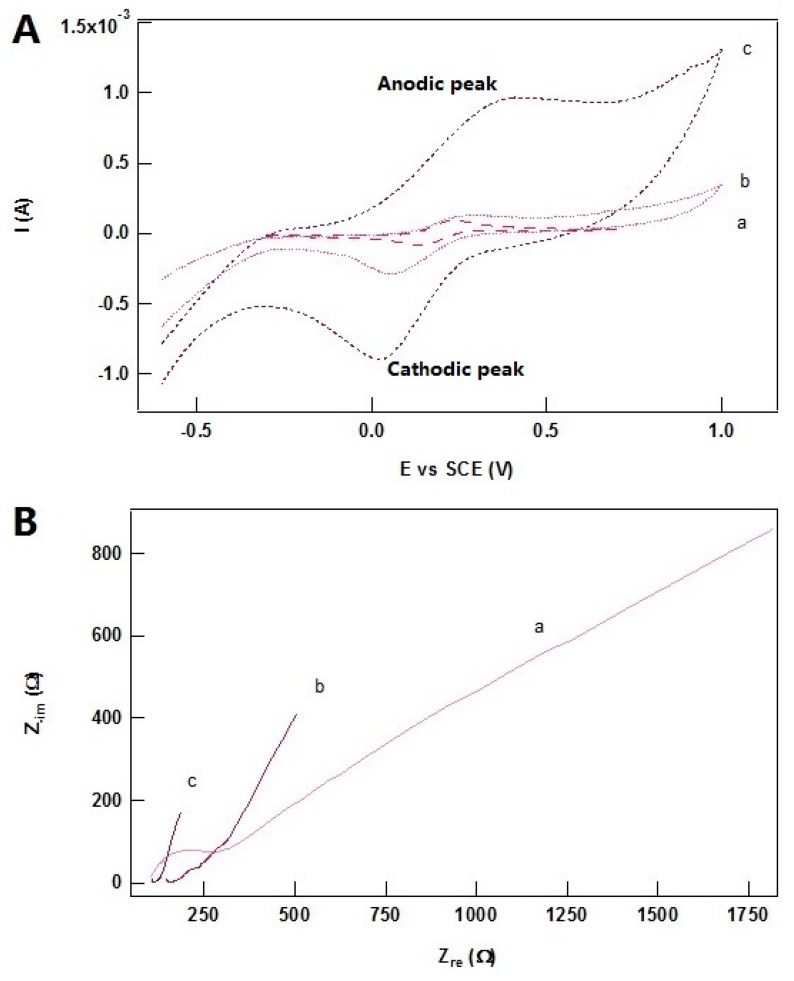
In (**A**), Cyclic voltammograms recorded in 5 mmol L^−1^ K_3_[Fe(CN)_6_]/K_4_[Fe(CN)_6_] solution containing 0.1 mol L^−1^ KCl at (a) GCE, (b) α-,γ-Fe/CNTs/CNOs buckypaper and (c) Fe_3_C/CNTs buckypaper produced in this work. Scan rate: 50 mV s^−1^. In (**B**), Nyquist diagrams obtained for (a) GCE, (b) α-,γ-Fe/CNTs/CNOs buckypaper and (c) Fe_3_C/CNTs in 5 mmol L^−1^ K_3_[Fe(CN)_6_]/K_4_[Fe(CN)_6_] solution containing 0.1 mol L^−1^ KCl.

The electrochemical properties of Fe_3_C/CNTs buckypapers produced in this work were then investigated by cyclic voltammetry (CV), at a scan rate of 50 mV using the K_3_[Fe(CN)_6_]/K_4_[Fe(CN)_6_] redox probe. In these measurements the properties of this buckypaper were compared to those of a glassy carbon electrode (GCE), and to those of another type of buckypaper obtained with the synthesis method in reference [21], and consisting of a layered structure comprising both Fe filled carbon nano-onions, (layer 1) and randomly oriented CNTs filled with α-,γ-Fe (layer 2). As can be seen in Figure 6, a significant peak current increase (*I*_p_) was observed for Fe_3_C/CNTs (curve c) in comparison to GCE and the other buckypaper (curve a and b). This phenomenon can be attributed to the possible intrinsic differences in (1) the structural arrangement of the buckypapers, and (2) the active surface areas in comparison to unmodified GCE. In particular, in the case of the buckypaper presented in this work (Fe_3_C-CNTs in Figure 6) the presence of a significant quantity of Sm particles on the top surface could possibly function as active sites for the electrochemical processes. 

Moreover, a well visible shift of cathodic and anodic peaks (Figure 6A) with the peak potential (Δ*E*_p_) separation of 214 and 387 mV for CNOs/α-,γ-Fe/CNTs, and Fe_3_C/CNTs buckypapers, respectively, can be noticed. The increase of peaks potentials separation indicates the absence of catalytic properties toward carried out electrochemical reaction. Interestingly, the cyclic voltammogram of Fe_3_C/CNTs possess a rectangular-like shape, demonstrating an electrochemical pseudo-capacitance behavior (resistive-capacitor), which is further investigated by electrochemical impedance spectroscopy (EIS) technique (Figure 6B). Most likely, this current is related to the charging of the double layer capacitance. Characteristic values obtained from CV measurements are collected in Table 1.

Accordingly, the EIS measurements were performed for the GC, CNOs-CNTs buckypaper, and Fe_3_C/CNTs buckypaper electrodes using the K_3_[Fe(CN)_6_]/K_4_[Fe(CN)_6_] redox probe in the presence of KCl. Figure 6B shows Nyquist plots of examined electrodes, where *Z*_re_ is the real part and *Z*_-im_ is the imaginary part of the complex impedance *Z*. Line (a) shown in Figure 6B, corresponds to EIS results obtained for unmodified GCE, where the semicircle diameter at higher frequencies corresponds to the charge transfer resistance (*R*_ct_), which controls the electron transfer kinetics of K_3_[Fe(CN)_6_]/K_4_[Fe(CN)_6_] at the electrode/electrolyte interface, meanwhile the resistance in the high frequency range (*R*_e_) is the resistance of the electrolyte, the contacts, and connections. The straight line is the impedance due to the mass transfer of the redox species to the electrode, described by Warburg. As can be seen, the EIS of the bare GCE is composed of a semi-circle and a straight line featuring a diffusion limiting step of the K_3_[Fe(CN)_6_]/K_4_[Fe(CN)_6_] probe.

In the case of the CNOs-CNTs buckypaper and Fe_3_C/CNTs buckypaper electrodes (Figure 6B, line b and c), the electron transfer kinetics of the redox probe becomes fast so that the semi-circle disappears and forms almost a straight line. The decrease of the charge transfer resistance is due to the higher electron transfer kinetics at the buckypaper/electrolyte interface, and is in agreement with CV results. A presence of a “knee-like” feature (Figure 6B, line b) can be observed in the Nyquist impedance plot for the CNOs-CNTs buckypaper electrode, which can be attributed to its complex structure characterized by two horizontal layers, of which the first one is composed of filled carbon CNOs structures and the second of randomly oriented CNTs. In the case of Fe_3_C/CNTs buckypaper (Figure 6B, line c) its impedance shows slight change, where the line is approaching the imaginary axis (*R_-_*_im_) in the low frequency region pointing out on the capacitive-like behaviour of the material. Due to our considerations, the enhanced capacitive properties of the Fe_3_C/CNTs buckypaper electrode, in comparison to that of CNOs-CNTs, can be assigned to the contribution from both the higher surface area of thicker CNTs layer, as well as to Fe and Sm catalyst particles present on the material layers and surface (for more details see EDX and SEM materials characterization). As it has been shown, the Fe particles not only act as a catalyst for the CNTs growth but at the same time contribute as the Fe_3_C/CNTs buckypaper modifier, which allow to achieve an enhanced capacitive behavior (together with the Sm particles) of the Fe_3_C/CNTs based material presented in this study. Furthermore, a slight deviation of the Nyquist plot from a straight line in the high frequency region is most probably attributed to charging processes of Fe_3_C/CNTs electrode.

## 4. Conclusions

In conclusion, we demonstrated an innovative CVD approach in which the large-scale fabrication of ferromagnetically-filled cm-scale buckypapers is achieved through the deposition of a mesoporous supported catalyst (SBA-16) on the used substrate. In particular, in this method, the deposition of the chosen catalyst on a SBA-16 support (on the used CVD substrate) has the crucial role of promoting the growth of CNTs with random orientation on a horizontal plane rather than in a vertical direction, therefore allowing a facile fabrication of cm-scale CNTs buckypapers with high purity. SBA-16 can be therefore considered not only as secondary catalyst source but also as growth-inhibitor, since it can prevent the growth of vertically aligned CNTs. The room temperature magnetic properties of the produced buckypapers were also investigated and revealed the presence of a high coercivity of 650 Oe. Additionally, the electrochemical properties of these buckypapers revealed an interesting pseudo-capacitor (resistive-capacitor) behaviour that makes the produced free-standing films suitable for applications in energy storage as flexible electrodes. Our measurements also suggest that the performances of these buckypapers are better than those present in other previously studied layered-buckypapers comprising Fe-filled CNTs obtained by pyrolysis of ferrocene or dichlorobenzene-ferrocene mixtures.

## Figures and Tables

**Table 1:** Cyclic voltammetry (CV) results obtained for GC, α-,γ-Fe/CNTs/CNOs electrodes and Fe_3_C/CNTs electrodes.

Electrode	*E*_C_ (mV)	*I*_C_ (A)	*E*_A_ (mV)	*I*_A_ (A)	Δ *E*_p_ (mV)
GCE	138	−6.358 × 10^−5^	237	1.020 × 10^−4^	99
CNOs-CNTs Buckypaper	55	−1.819 × 10^−4^	269	1.507 × 10^−4^	214
Fe_3_C/CNTs Buckypaper	19	−3.788 × 10^−4^	397	9.386 × 10^−4^	387

## Supplementary Materials

The following are available online at www.mdpi.com/1996-1944/10/10/1216/s1, Figure S1: Statistical investigation of the outer diameter of the Fe_3_C-filled CNTs comprised in the buckypaper produced by using SBA-16 as growth promoter. Note that the average diameter was 79.2 nm, while the length of the CNTs (as evaluated in the SEM micrographs presented in the manuscript) was estimated to be in the order of many tens of micrometres (i.e., 50–100 micrometres).

## References

[1] Iijima S. (1991). Helical Microtubules of Graphite Carbon. Nature.

[2] Iijima S., Ichihashi T. (1993). Single-shell Carbon Nanotubes of 1-nm Diameter. Nature.

[3] Dresselhaus M.S., Dresselhaus G., Avouris P. (2001). Carbon Nanotubes: Synthesis, Structure, Properties and Applications.

[4] Sitharaman B., Wilson L.J. (2006). Gadonanotubes as New Highperformance MRI Contrast Agents. Int. J. Nanomed..

[5] Hampel S., Leonhardt A., Selbmann K., Biedermann D., Elefant D., Muller C., Gemming T., Büchner B. (2006). Growth and Characterization of Filled Carbon Nanotubes with Ferromagnetic Properties. Carbon.

[6] Leonhardt A., Ritschel M., Elefant D., Mattern N., Biedermann K., Hampel S., Müller C., Gemming T., Büchner B. (2005). Enhanced Magnetism in Fe-filled Carbon Nanotubes Produced by Pyrolysis of Ferrocene. J. Appl. Phys..

[7] Leonhardt A., Ritschel M., Kozhuharova R., Graff A., Muhl T., Huhle R., Elefant D., Schneider C.M. (2003). Synthesis and Properties of Filled Carbon Nanotubes. Diam. Relat. Mater..

[8] Terrones H., López-Urías F., Muñoz-Sandoval E., Rodríguez-Manzo J.A., Zamudio A., Elías A.L., Terrones M. (2006). Magnetism in Fe-based and Carbon Nanostructures: Theory and Applications. Solid State Sci..

[9] Boi F.S., Maugeri S., Guo J., Lan M., Wang S., Wen J., Mountjoy G., Baxendale M., Nevill G., Wilson R.M. (2014). Controlling the Quantity of α-Fe Inside Multiwall Carbon Nanotubes Filled with Fe-based Crystals: The Key Role of Vapor Flow-Rate. Appl. Phys. Lett..

[10] Muller C., Hampel S., Elefant D., Biedermann K., Leonhardt A., Ritschel M., Büchner B. (2006). Iron Filled Carbon Nanotubes Grown on Substrates with Thin Metal Layers and Their Magnetic Properties. Carbon.

[11] Muller C., Golberg D., Leonhardt A., Hampel S., Buchner B. (2006). Growth studies, TEM and XRD Investigations of Iron-Filled Carbon Nanotubes. Phys. Status Solidi A.

[12] Morelos-Gomez A., Lopez-Urias F., Munoz-Sandoval E., Dennis C.L., Shull R.D., Terrones H., Terrones M. (2010). Controlling High Coercivities of Ferromagnetic Nanowires Encapsulated in Carbon Nanotubes. J. Mater. Chem..

[13] Guo J., He Y., Wang S., Boi F.S. (2016). Mapping the Transition from Free-Standing Vertically-Aligned Fe3C-filled Carbon Nanotube Films to Entangled Randomly-Oriented Carbon Nanotube Buckypapers in Presence of a Great Excess of Ferrocene. Carbon.

[14] Boi F.S., Guo J., Wang S., He Y., Xiang G., Zhang X., Baxendale M. (2016). Fabrication of Cm Scale Buckypapers of Horizontally Aligned Multiwall Carbon Nanotubes Highly Filled with Fe_3_C: The Key Roles of Cl and Ar-Flow Rate. Chem. Commun..

[15] Wang W., Wang K., Lv R., Wei J., Zhang X., Kang F., Chang J., Shu Q., Wang Y., Wu D. (2007). Synthesis of Fe-Filled Thin-Walled Carbon Nanotubes with High Filling Ratio by Using Dichlorobenzene as Precursor. Lett. Ed. Carbon.

[16] Lv R., Kang F., Wang W., Wei J., Gu J., Wang K., Wu D. (2007). Effect of Using Chlorine-Containing Precursors in the Synthesis of FeNi-Filled Carbon Nanotubes. Carbon.

[17] Lv R., Cao A., Kang F., Wang W., Wei J., Gu J., Wang K., Wu D. (2007). Single-Crystalline Permalloy Nanowires in Carbon Nanotubes: Enhanced Encapsulation and Magnetization. J. Phys. Chem. C.

[18] Lv R., Tsuge S., Gui X., Takai K., Kang F., Enoki T., Wei J., Gu J., Wang K., Wu D. (2009). In Situ Synthesis and Magnetic Anisotropy of Ferromagnetic Buckypaper. Carbon.

[19] Gui X., Wang K., Wang W., Wei J., Zhang X., Lv R., Jia Y., Shu Q., Kang F., Wu D. (2009). The Decisive Roles of Chlorine-Contained Precursor and Hydrogen for the Filling Fe Nanowires into Carbon Nanotubes. Mater. Chem. Phys..

[20] Lv R., Kang F., Gu J., Gui X., Wei J., Wang K., Wu D. (2008). Carbon Nanotubes Filled with Ferromagnetic Alloy Nanowires: Lightweight and Wide-Band Microwave Absorber. Appl. Phys. Lett..

[21] Boi F.S., Hu Y., Wang S., He Y. (2016). Controlling High Coercivities in Cm-Scale Buckypapers with Unusual Stacking of Vertically Aligned and Randomly Entangled Fe-filled Carbon Nanotubes. RSC Adv..

[22] Boi F.S., Guo J., Lan M., Xiang G., Wang S., Wen J., Zhang S. (2015). Synthesis of Planar-Graphite Structures with Embedded Fe(x)Pd(x) or CoPd-CoPd_2_ Phases and of Carbon Nanotubes Filled with Fe(x)Pd(x) with Variable Filling Ratio. Carbon.

[23] Rivera-Munoz E.M., Huarache-Acuna R. (2010). Sol Gel-Derived SBA-16 Mesoporous Material. Int. J. Mol. Sci..

[24] Li L., King D.L., Liu J., Huo Q., Zhu K., Wang C., Gerber M., Stevens D., Wang Y. (2009). Stabilization of Metal Nanoparticles in Cubic Mesostructured Silica and Its Application in Regenerable Deep Desulfurization of Warm Syngas. Chem. Mater..

[25] Dervishi E., Li Z., Xu Y., Saini V., Watanabe F., Biris A.R., Bonpain A., Garbay J.J., Meriet A., Richard M. (2009). The Influence of Fe-Co/MgO Catalyst Composition on the Growth Properties of Carbon Nanotubes. Particul. Sci. Technol..

[26] Carta D., Boi F., Corrias A., Bullita S., Konya Z., Casula M.F. (2014). Iron/cobalt-SBA-16 cubic mesoporous composites as catalysts for the production of multi-walled carbon nanotubes. J. Porous Mater..

[27] Carta D., Bullita S., Casula M.F., Casu A., Falqui A., Corrias A. (2013). Cubic Mesoporous Silica (SBA-16) Prepared Using Butanol as the Co-Surfactant: A General Matrix for the Preparation of FeCo-SiO_2_ Nanocomposites. ChemPlusChem.

[28] Kleitz F., Solovyov L.A., Anilkumar G.M., Choi S.H., Ryoo R. (2004). Transformation of Highly Ordered Large Pore Silica Mesophases (*Fm*3*m*, *Im*3*m* and *p*6*mm*) In a Ternary Triblock Copolymer-Butanol-Water System. Chem. Commun..

[29] Larson A.L., Von Dreele R. (1986). GSAS, General Structure Analysis System Report LAUR 86–748.

[30] Lee C.-J., Park J. (2000). Growth Model of Bamboo-Shaped Carbon Nanotubes by Thermal Chemical Vapor Deposition. Appl. Phys. Lett..

[31] Murakami H., Hirakawa M., Tanaka C., Yamakawa H. (2000). Field Emission from Well-Aligned, Patterned, Carbon Nanotube Emitters. Appl. Phys. Lett..

[32] Boi F., Wang S., He Y. (2016). Mapping the Transition from Catalyst-Pool to Bamboo-Like Growth-Mechanism in Vertically-Aligned Free-Standing Films of Carbon Nanotubes Filled with Fe_3_C: The key Role of Water. AIP Adv..

[33] Rossella F., Mozzati M.C., Bordonali L., Lascialfari A., Soldano C., Ortolani L., Bellani V. (2016). Nanostructured magnetic metamaterials based on metal-filled carbon nanotubes. Carbon.

[34] Sahoo P., Djieutedjeu H., Poudeu P.F.P. (2013). Co_3_O_4_ nanostructures: The effect of synthesis conditions on particles size, magnetism and transport properties. J. Mater. Chem. A.

