# Supplementary material for: Cl-Assisted Large Scale Synthesis of Cm-Scale Buckypapers of Fe3C-Filled Carbon Nanotubes with Pseudo-Capacitor Properties: The Key Role of SBA-16 Catalyst Support as Synthesis Promoter
Source: Materials (Basel). 2017 Oct 23;10(10):1216. doi: 10.3390/ma10101216 (PMC5667022; doi:10.3390/ma10101216)
Supplement: Supplementary file 1 [file materials-10-01216-s001.pdf]

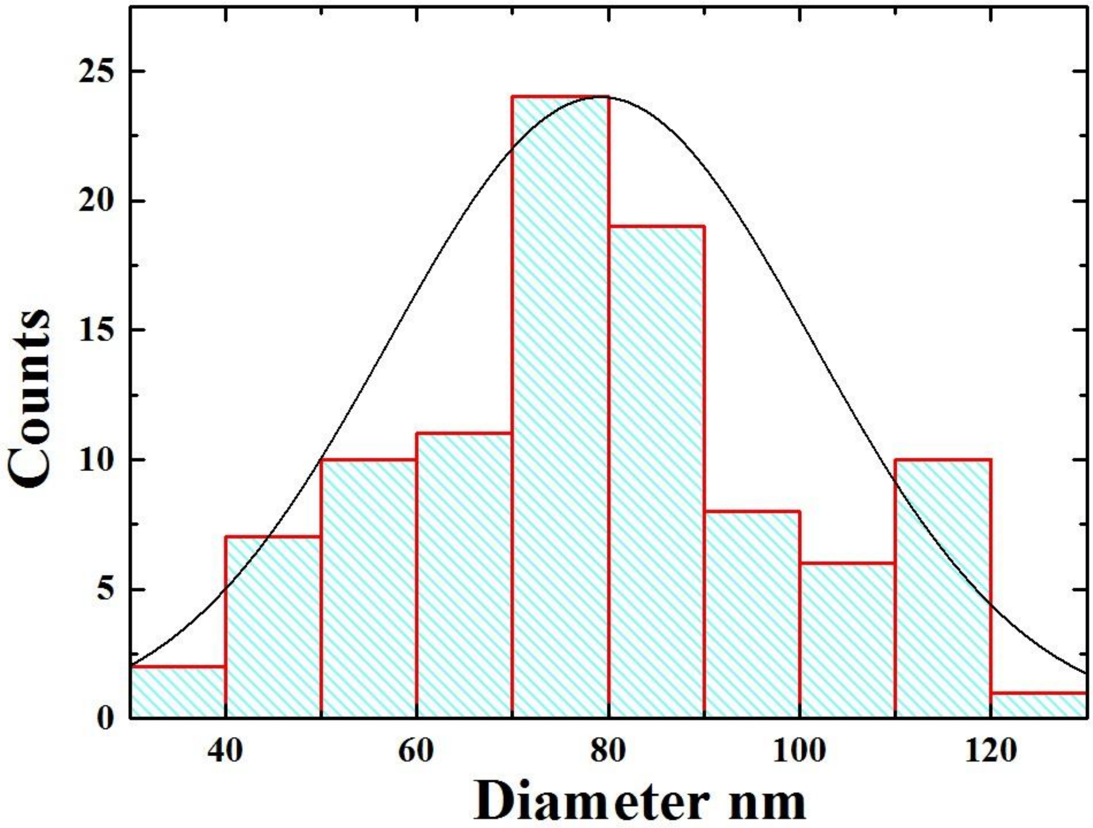

Figure S1. Statistical investigation of the outer diameter of the Fe_3C-filled CNTs comprised in the buckypaper produced by using SBA-16 as growth promoter. Note that the average diameter was 79.2 nm, while the length of the CNTs (as evaluated in the SEM micrographs presented in the manuscript) was estimated to be in the order of many tens of micrometres (i.e 50–100 micrometres).

© 2017 by the authors. Submitted for possible open access publication under the terms and conditions of the Creative Commons Attribution (CC-BY) license (http://creativecommons.org/licenses/by/4.0/).
